# New Complementary Resonator for Permittivity- and Thickness-Based Dielectric Characterization

**DOI:** 10.3390/s23229138

**Published:** 2023-11-12

**Authors:** Tanveerul Haq, Slawomir Koziel

**Affiliations:** 1 Engineering Optimization and Modeling Center, Reykjavik University, 102 Reykjavik, Iceland; koziel@ru.is; 2 Faculty of Electronics, Telecommunications and Informatics, Gdansk University of Technology, 80-233 Gdansk, Poland

**Keywords:** complementary crossed arrow resonator, calibration, design optimization, dielectric characterization, high sensitivity, inverse modeling, permittivity, thickness

## Abstract

The design of high-performance complementary meta-resonators for microwave sensors featuring high sensitivity and consistent evaluation of dielectric materials is challenging. This paper presents the design and implementation of a novel complementary resonator with high sensitivity for dielectric substrate characterization based on permittivity and thickness. A complementary crossed arrow resonator (CCAR) is proposed and integrated with a fifty-ohm microstrip transmission line. The CCAR’s distinct geometry, which consists of crossed arrow-shaped components, allows for the implementation of a resonator with exceptional sensitivity to changes in permittivity and thickness of the material under test (MUT). The CCAR’s geometrical parameters are optimized to resonate at 15 GHz. The CCAR sensor’s working principle is explained using a lumped-element equivalent circuit. The optimized CCAR sensor is fabricated using an LPKF protolaser on a 0.762-mm thick dielectric substrate AD250C. The MUTs with dielectric permittivity ranging from 2.5 to 10.2 and thickness ranging from 0.5 mm to 1.9 mm are used to investigate the properties and calibrate the proposed CCAR sensor. A two-dimensional calibration surface is developed using an inverse regression modelling approach to ensure precise and reliable measurements. The proposed CCAR sensor is distinguished by its high sensitivity of 5.74%, low fabrication cost, and enhanced performance compared to state-of-the-art designs, making it a versatile instrument for dielectric characterization.

## 1. Introduction

The versatility and unique features of microwave sensors, such as fast response times, extensive sensing ranges, and compatibility with a wide range of climate conditions, make them essential tools in various kinds of industries such as agriculture, automotive, biomedicine, communication, and manufacturing [1]. Metamaterial structures can boost the efficacy of microwave sensors by enhancing their sensitivity, selectivity, and compactness. Due to their artificial nature, metamaterials provide unique design flexibility in terms of frequency modulation and miniaturization [2]. The properties of metamaterial structures can be controlled by adjusting their geometrical parameters, such as shape, size, and periodicity. This adaptability permits customization of frequency and application-specific responses [3]. Split ring resonators (SRR) [4] and complementary split ring resonators (CSRR) [5] are utilized extensively in numerous metamaterial-based microwave sensors and devices, such as filters [6], antennas [7], absorbers [8], and radars [9]. Their distinctive characteristics, including resonant behavior, magnetic response, and negative permeability, make them valuable components for augmenting the performance of microwave sensors. SRR-based microwave sensors are often magnetically connected to the microstrip transmission line (MTL). Magnetic coupling of SRR has been accomplished by etching the SRR geometries on the top layer of the microwave sensor near the MTL [10]. This configuration enables effective coupling between the SRR and the MTL, allowing the SRRs to resonate and generate an electric field near the narrow split zone. This intensified electric field has been utilized for liquid characterization [11] and biomedical sensing [12]. The limited and restricted sensing area of SRR-based microwave sensors makes them not optimal for the purpose of microwave sensing of sizable samples. The resolution of this matter has been achieved by the utilization of CSRR in replacement of SRR for the assessment of sizeable dielectric specimens [13,14,15].

In [13], a complementary circular spiral resonator-based microwave sensor with a resonant frequency of 2.29 GHz has been used for dielectric characterization of Teflon, quartz glass, FR-4 epoxy, and silicon nitride. The maximum relative sensitivity of 3.57 percent has been achieved due to interaction with the Teflon material with dimensions 27 mm × 6 mm × 1 mm and a relative permittivity (*ε_r_*) of 2.1. In [14], the dielectric characterization of RT5880, RO4003, FR-4, and RO6010 has been performed using a CSRR-based microwave sensor with a resonant frequency of 2.7 GHz. Due to interaction with an RT5880 with dimensions of 40 mm × 25 mm × 0.5 mm and *ε_r_* of 2.2, the maximum relative sensitivity of 4.01 percent has been attained. In [15], a microwave sensor based on a novel complementary curved-ring resonator (CCRR) with a resonant frequency of 3.49 GHz has been used for dielectric characterization of AD255, AD300, RO4535, and FR4. The maximum relative sensitivity of 5.31 percent has been achieved due to interaction with an AD255 material with dimensions 15 mm × 15 mm × 1.5 mm and a relative permittivity of 2.55. Measuring permittivity and thickness is critical in practical applications for precisely characterizing dielectric materials in various electrical and microwave devices. A few microwave sensors have been proposed to characterize thickness and permittivity simultaneously [16,17]. In [16], a single-compound complementary split ring resonator has been proposed to determine the thickness and *ε_r_* of dielectric substrates using the inverse square resonance frequency method. To determine both parameters (thickness and relative permittivity) of the MUTs, the suggested approach requires two resonant frequencies, and the maximum measurement error is 10.9%. In [17], a dual-notch resonator has been employed to determine the relative permittivity and thickness of dielectric materials (TLY5 and RO4350) using the curve-fitting technique. The maximum measurement error with this technique is 12.07%, and it requires solving two equations to determine thickness and permittivity concurrently. The previously described microwave sensors have a sensitivity limitation of up to five percent and a significant measurement error of more than ten percent. Both of these limitations affect the accuracy of the sensors’ findings. Customizing microwave sensors for particular applications and attaining precise measurements requires meticulous design of novel resonant structures and their accurate calibration.

This work presents a novel complementary resonator with excellent sensitivity, low cost, and improved performance. A complementary crossed arrow resonator (CCAR) is proposed, which is connected to an MTL to provide a high-sensitivity sensor. The CCAR’s geometric parameters are tuned to resonate at 15 GHz. The optimized CCAR sensor is used for measuring dielectric materials within the range of *ε_r_* from 2.5 to 10.2, and thickness from 0.5 mm to over 2.0 mm. Its maximum sensitivity exceeds five percent. A calibration surface based on the inverse regression model is developed to make the proposed structure a robust and reliable tool for predicting the electromagnetic properties of various MUTs with a maximum measurement error of less than eight percent.

The subsequent sections of the paper are arranged as follows. In Section 2, the CCAR sensor’s design geometry and lumped element circuit model are introduced. The fabrication and measurements of the optimized CCAR sensor are the focus of Section 3. In Section 4, the calibration procedure utilizing the inverse regression modeling approach is described in detail, followed by verification experiments and comparison to literature-reported state-of-the-art devices. The work concludes with Section 5.

## 2. Sensor Design

**Figure 1:**
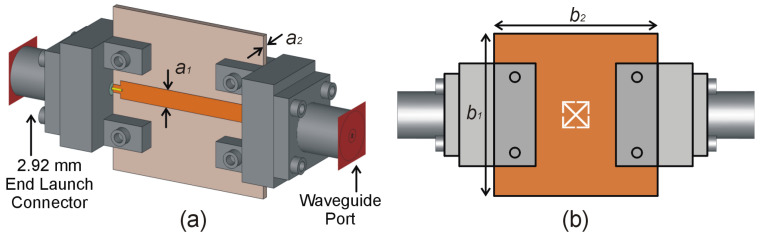
Proposed CCAR-based sensor: (**a**) excitation of the proposed sensor, (**b**) bottom view of the sensor with complementary crossed arrows resonator.

**Figure 2:**
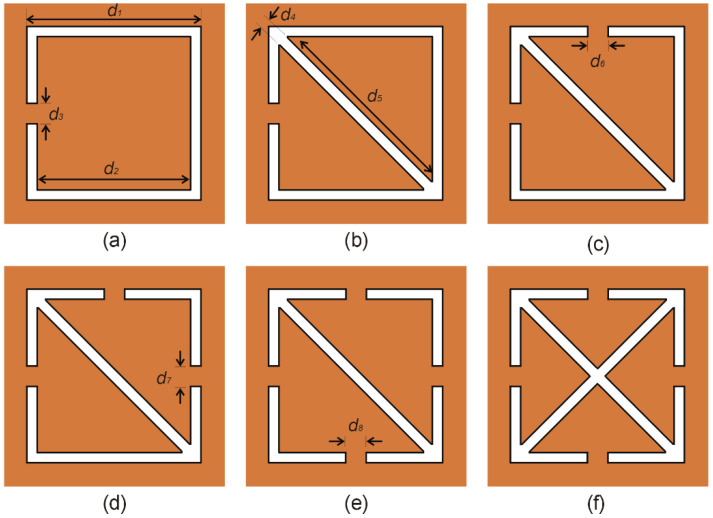
The evolution of the CCAR geometry from the CSSRR, (**a**) CSSRR (**b**) first modification (M1), (**c**) second modification (M2), (**d**) third modification (M3), (**e**) fourth modification (M4), (**f**) CCAR.

**Figure 3:**
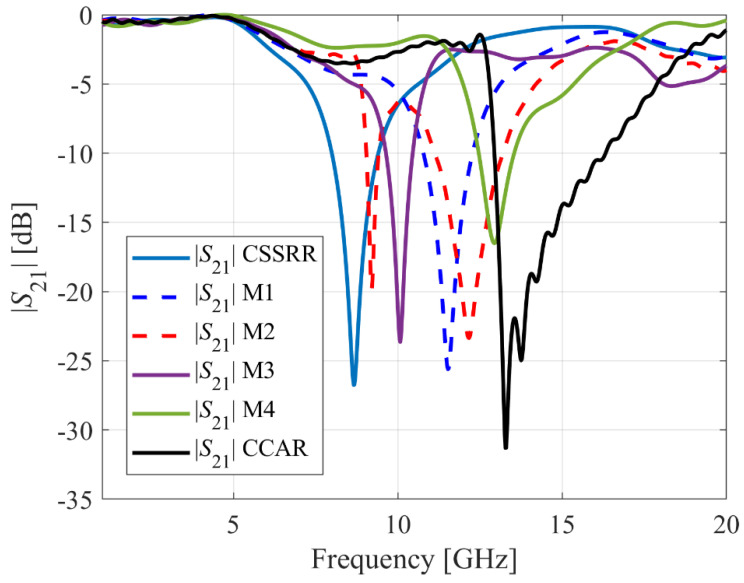
The transmission response of the sensor influenced by each modification.

The proposed sensor based on a complementary crossed arrows resonator (CCAR) linked to a fifty-ohm microstrip transmission line (MTL), is discussed in this section. The MTL of the width *a*_1_ = 2.192 mm is printed on the upper layer of the AD250C substrate of a square shape and size *b*_1_ = 20 mm and *b*_2_ = 20 mm, as shown in Figure 1a. The geometry of the CCAR is derived from the fundamental structure known as the complementary square split ring resonator (CSSRR), which has been used recently to design negative group delay circuit [18], substrate-integrated waveguide filter [19], wideband antenna [20], and microwave sensor [21]. Figure 2 shows the progression of the CCAR geometry from its predecessor, the CSSRR. Initially, a CSSRR with the three geometric parameters (*d*_1_ = 4 mm, *d*_2_ = 3 mm and *d*_3_ = 0.5 mm) is designed, as shown in Figure 2a. The initial geometric parameters were chosen by a comprehensive analysis of the existing literature [18,19,20,21]. After simulation, the CSSRR under investigation exhibits a resonant frequency of 8.6 GHz, accompanied by a notable notch depth of −26.7 dB. In first modification (M1), a cross line with the two geometric parameters (*d*_4_ = 0.28 mm, and *d*_5_ = 3.96 mm) is introduced in the CSSRR, as shown in Figure 2b. Following the simulation, M1 gives a resonant frequency of 11.5 GHz with a notch depth of −25.6 dB. In the second modification (M2), an additional split (*d*_6_ = 0.5 mm) is integrated into the M1, as depicted in Figure 2c. Following the simulation, M2 exhibits a twin-notch resonant frequency, initially at 9.1 GHz with a notch depth of −19.5 dB, and subsequently at 12.6 GHz with a notch depth of −23.3 dB. The third modification (M3) introduces a third split (*d*_7_ = 0.5 mm) in the M2, as shown in Figure 2d. M3 produces a single resonance frequency of 10.07 GHz with a notch depth of −23.6 dB after simulation. As seen in Figure 2e, the fourth modification (M4) adds a fourth split (*d*_8_ = 0.5 mm) to the M3. After simulation, M4 yields a single resonance frequency of 12.9 GHz with a notch depth of −16.5 dB. To produce the CCAR, the last modification adds another cross line with the same dimension as the first one to the M4, as shown in Figure 2f. Following the simulation, CCAR exhibits a twin notch resonant frequency, initially at 13.2 GHz with a notch depth of −31.3.5 dB, and subsequently at 13.7 GHz with a notch depth of −24.9 dB. The effect of each modification on the sensor’s transmission response is shown in Figure 3.

To optimize the sensor’s performance, the geometric parameters of the CCAR structure are aggregated into a vector ***x*** = [*d*_1_ *d*_2_ *d*_3_ *d*_4_ *d*_5_ *d*_6_ *d*_7_ *d*_8_]*^T^*. The fundamental resonant frequency is designated as *f*_0_, and the level of the |*S*_21_| at *f*_0_ is given as *L*_0_.

The task of design optimization is defined as [22]

(1) $$x^{*}=\arg\underset{x}{\min}U\left(x,f_{t}\right)$$

where the variable x* represents the optimal design to be determined, whereas *f_t_* denotes the desired notch frequency.

The goal of the function is formally defined as

(2) $$U\left(x,f_{t}\right)=L_{0}\left(x\right)+\beta {\left(f_{t}-f_{0}\left(x\right)\right)}^{2}$$

**Figure 4:**
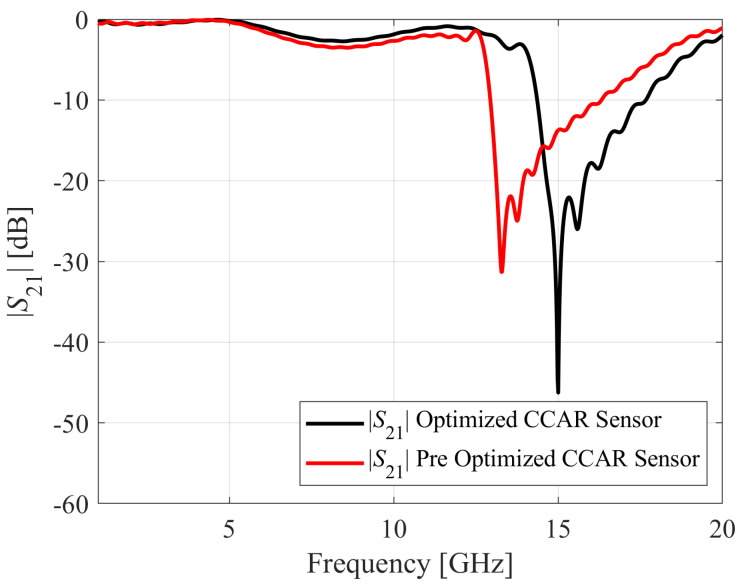
Microwave transmission coefficients *S*_21_ simulated for the pre-optimized and optimized CCAR-based design.

The first variable in (2) corresponds to the principal objective, which is the enhancement of the notch depth. Conversely, the subsequent component serves as a penalty function employed to ensure the allocation of the resonant frequency at *f_t_*. The optimization process of the CCAR parameters leads to *d*_1_ = 3.74 mm, *d*_2_ = 2.94 mm, *d*_3_ = 0.20 mm, *d*_4_ = 0.28 mm, *d*_5_ = 4.48 mm, *d*_6_ = 0.20 mm, *d*_7_ = 0.20 mm, and *d*_8_ = 0.20 mm. The *f*_0_ of the optimized sensor is 15 GHz with a notch depth of −46.25 dB. The optimized sensor has a more precisely assigned *f*_0_ and increased notch depth as compared to the pre-optimized sensor, cf. Figure 4.

**Figure 5:**
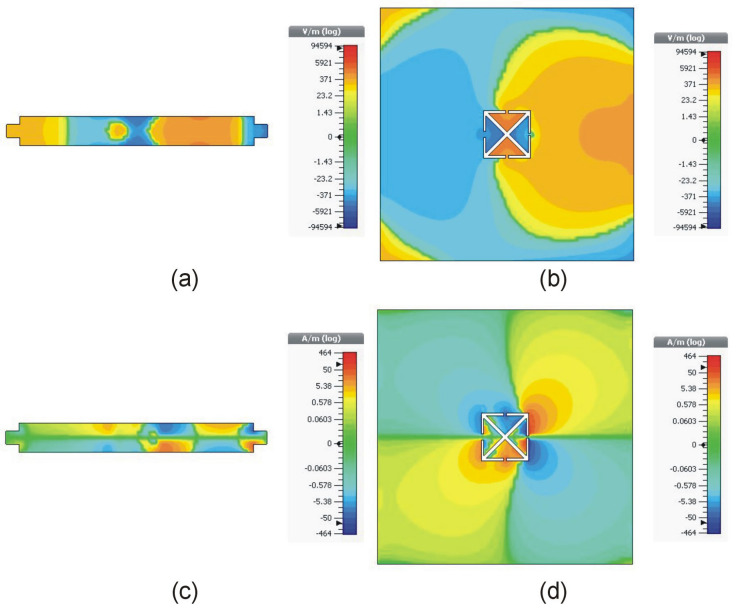
Distribution of electromagnetic fields for the optimized sensor at the resonant frequency (15 GHz) (**a**) *E* field of MTL, (**b**) *E* field of CCAR, (**c**) *H* field of MTL, (**d**) *H* field of CCAR.

For sensitivity analysis regarding structural evolution, a substrate material AD250C with constant dimensions of 7 mm × 7 mm × 0.762 mm is placed within the sensor’s ground plane. Table 1 presents the unloaded quality factor of the sensor, as well as the changes in resonant frequency and amplitude variation resulting from the sensor’s interaction with the material under test (MUT). As the geometry of the resonator undergoes changes from CSSRR to CCAR, the resonant frequency increases from 8.6 GHz to 15 GHz, while the frequency shift increases from 1.2 GHz to 2.4 GHz. The data reported in Table 1 illustrates that the optimized CCAR sensor exhibits a frequency shift that is twice as large as that of the CSSRR. The sensor’s higher resonance frequency, high quality factor, and increased electromagnetic field concentration are the primary causes of the enhanced frequency shift. The electric and magnetic fields of the MTL and optimized CCAR at the resonant frequency of the sensor are shown in Figure 5.

**Figure 6:**
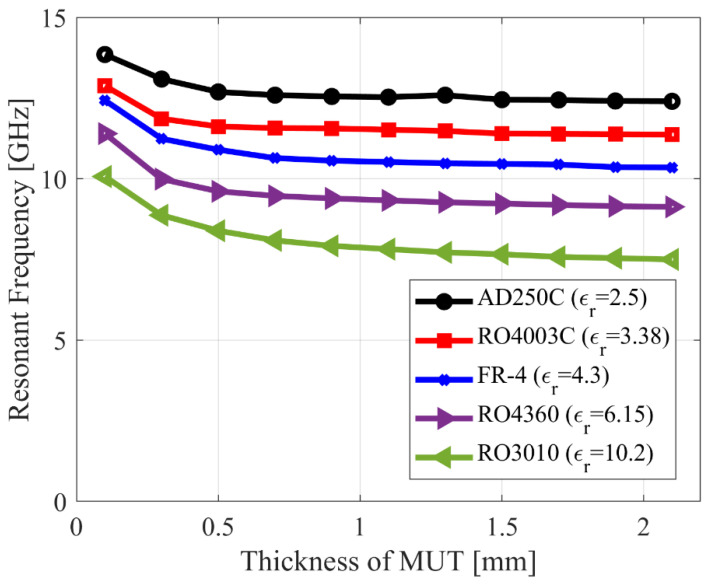
Effect of thickness of material under test on the resonant frequency of the optimized CCAR-sensor.

Five MUTs of constant size (5 mm × 5 mm) are positioned on the optimized CCAR sensor to investigate the individual influence of MUT thickness. Figure 6 depicts the results of increasing the thickness of each MUT from 0.1 mm to 2.1 mm. The CCAR’s resonant frequency decreases as the thickness of each MUT increases from 0.1 mm to 2.1 mm. The capacitance of the CCAR structure is affected by the increase in MUT thickness. The addition of a thicker MUT causes a rise in capacitance. The CCAR structure’s resonance frequency is reduced due to the reduction in its overall electrical length produced by the addition of extra capacitance.

## 3. Fabrication and Experimental Validation

**Figure 7:**
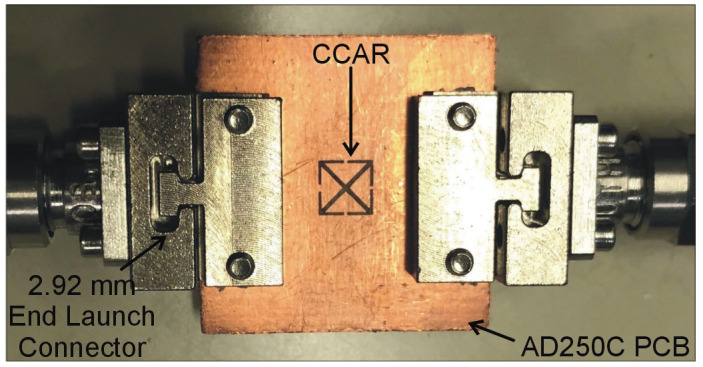
Fabricated prototype of the proposed sensor based on complementary crossed arrows resonator (CCAR).

**Figure 8:**
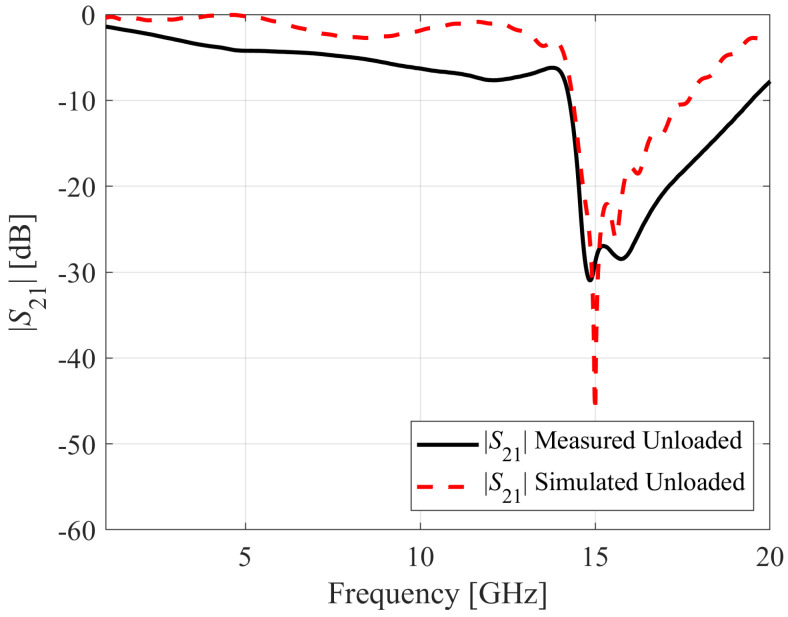
A comparison between simulated and measured microwave transmission coefficients *S*_21_ for the unloaded CCAR sensor.

This section investigates the fabrication process of the optimized sensor and the subsequent measurements conducted using selected dielectric samples. The optimized CCAR sensor has been fabricated using the LPKF Protolaser on an AD250C printed circuit board (PCB), cf. Figure 7. The LPKF Protolaser machine employs laser technology, specifically a scanner-guided laser operating at a wavelength of 355 nm within the ultraviolet (UV) spectrum. This laser is utilized to selectively eliminate material, such as copper (in our case 18 µm), from the surface of a PCB in order to generate the sensor layouts. The dimensions of the constructed prototype are consistent with those outlined in the previous section. The sensor is linked to the Anritsu MS4644B vector network analyzer through the utilization of 2.92 mm end-launch connectors in order to measure the transmission coefficients *S*_21_. Figure 8 presents a comparison between the simulated and measured *S*_21_ values for the optimized sensor. The optimized CCAR sensor’s simulated and measured resonant frequencies are 15 GHz and 14.85 GHz, with notch depths of −46.25 dB and −30.97 dB, respectively.

The observed deviation of 0.15 GHz between the resonant frequencies obtained from simulation and measurement can be related to fabrication tolerances, encompassing factors such as changes in dimensions, alignment, and positioning. The observed mismatch of −15.28 dB between the simulated and measured notch depths can be attributed to various factors, including material characteristics, surface roughness, and substrate losses.

**Figure 9:**
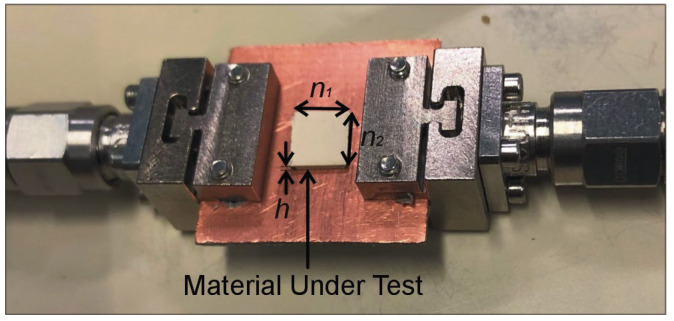
The experimental configuration of the fabricated CCAR sensor for testing of the material under test (MUT).

**Figure 10:**
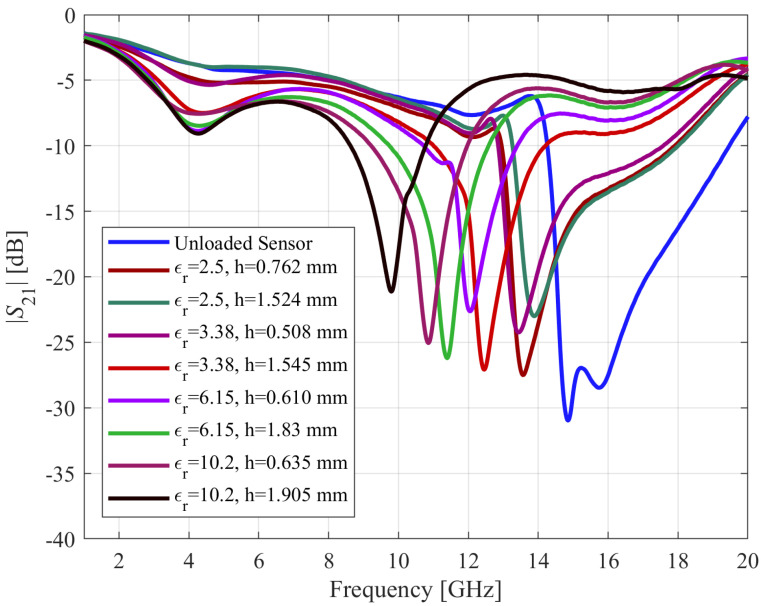
Transmission coefficients *S*_21_ of the fabricated CCAR sensor measured due to interaction with dielectric materials featuring various permittivity and thickness.

Four materials with known *ε_r_* values, including *ε_r_* = 2.5 (AD250C), *ε_r_* = 3.38 (RO4003), *ε_r_* = 6.15 (R04360), and *ε_r_* = 10.2 (RO3010) are used as material under test (MUT) for dielectric characterization. It is critical that that the size of the MUT be greater than the size of the resonator in order for it to interact appropriately with the electromagnetic field emitted by the CCAR. As the external length of the optimized CCAR is 3.74 mm, the sample dimensions are set to *n*_1_ = 5 mm and *n*_2_ = 5 mm, while the thickness *h* varies from 0.5 mm to 1.9 mm, cf. Figure 9. Figure 10 illustrates the initial transmission response of the CCAR sensor resulting from its interaction with the eight MUTs. The relative sensitivity of the proposed CCAR sensor can be computed as [22]:

(3) $$S_{\varepsilon_{r}}=\frac{f_{u}-f_{l}}{f_{l}\left(\varepsilon_{r}-1\right)}\times 100$$

where *f_u_* = 14.85 GHz is the unloaded resonant frequency of the CCAR microwave sensor, and *f_l_* is the resonant frequency of the CCAR sensor while interacting with the sample under test. In the case of the AD250C sample, characterized by a relative permittivity *ε_r_* of 2.5 and a thickness of 0.762 mm, the resonant frequency *f_l_* is determined to be 13.57 GHz. This resonant frequency corresponds to a maximum sensitivity of 5.74%. In the subsequent part of this section, a portion of the collected data will be utilized for the purpose of calibrating the fabricated CCAR sensor.

## 4. Sensor Calibration

This section discusses a methodology for calibrating the CCAR sensor by utilizing measurement data acquired from a collection of samples with predetermined attributes, specifically the relative permittivity *ε_r_* and thickness *h*. The provided data is utilized to develop an inverse regression model, which enables direct predictions of the permittivity of the material under test (MUT). These predictions are based on the measured resonant frequency of the CCAR sensor when loaded with the sample, as well as the measured thickness of the sample. 

As mentioned earlier, the resonant frequency of the CCAR sensor is affected by both the permittivity *ε_r_* and height *h* of the sample. Consequently, the calibration model must consider both of these variables. Given the marginal nonlinearity, we assume the following analytical form for the calibration model:

(4) $$\varepsilon_{r}=F(f_{0},h,a)=a_{0}+a_{1}f_{0}+a_{2}f_{0}^{2}+a_{3}h+a_{4}h^{2}$$

where the variable *f*_0_ represents the resonant frequency of the CCAR sensor as measured when it interacts with the MUT of thickness *h*, whereas ***a*** = [*a*_0_ *a*_1_ *a*_2_ *a*_3_ *a*_4_]*^T^* is a vector of model coefficients. Note that the model is linear with respect to *h*, which is sufficient given that the effects of sample thickness are much less pronounced than the effects of permittivity alteration.

The model yields the predicted permittivity *ε_r_* of the MUT. Identification of model coefficients is achieved by solving linear regression problems

(5) $$\varepsilon_{r.j}=F(f_{0.j},h_{j},a),\;\;\;\;j=1,\ldots ,N$$

Therein, *ε_r.j_* represents the actual relative permittivity of the *j*th calibration sample, *f*_0.*j*_ represents the measured resonant frequency of the sensor due to interaction with the *j*th sample; the thickness of the *j*th sample used for calibration purposes is represented by *h_j_*. *N* represents the total number of the calibration samples. The coefficients ***a*** are found by minimizing the error function

(6) $$E(a)=\left\|\left[\begin{array}{c} \varepsilon_{r.1}\\ \vdots \\ \varepsilon_{r.N} \end{array}\right]-\left[\begin{array}{c} F(f_{0.1},h_{1},a)\\ \vdots \\ F(f_{0.N},h_{N},a) \end{array}\right]\right\|$$

Because the regression problems (5) are linear with respect to the model coefficients, the least-square solution can be found analytically as

(7) $$a={\left[A^{T}A\right]}^{-1}A^{T}\left[\begin{array}{c} \varepsilon_{r.1}\\ \vdots \\ \varepsilon_{r.N} \end{array}\right]$$

where

(8) $$A=\left[\begin{array}{ccc} 1 & f_{0.1} & f_{0.1}^{2}\;\;\;h_{1}^{}\;\;\;h_{1}^{2}\\ \vdots & \vdots & \vdots \;\;\;\;\;\;\vdots \;\;\;\;\;\vdots \\ 1 & f_{0.N} & f_{0.N}^{2}\;\;\;h_{N}\;\;\;h_{N}^{2} \end{array}\right]$$

It should be emphasized that linearity of the calibration model with respect to its input parameters must not be mistaken with linearity of the underlying regression problem. The calibration model is not linear with respect to *f*_0_ and *h*; however, it is linear with respect to the model coefficients. In other words, it can be written as *ε_r_*(*f*_0_,*h*;***a***) = [*a*_0_ *a*_1_ *a*_2_ *a*_3_ *a*_4_]·[*v*_1_(*f*_0_,*h*) *v*_2_(*f*_0_,*h*) *v*_3_(*f*_0_,*h*) *v*_4_(*f*_0_,*h*) *v*_5_(*f*_0_,*h*)]*^T^*, where the basis functions are *v*_1_(*f*_0_,*h*) = 1, *v*_2_(*f*_0_,*h*) = *f*_0_, *v*_3_(*f*_0_,*h*) = *f*_0_^2^, *v*_4_(*f*_0_,*h*) = *h*, *v*_5_(*f*_0_,*h*) = *h*^2^.

In this study, a total of eight calibration samples are utilized. The measurement has been repeated ten times for each sample, and the average value of the resonant frequency, accompanied by its standard deviation as an indicator of error, has been recorded. The pertinent data has been compiled and presented in Table 2.

The model coefficients calculated using (7) and the data in Table 2 are ***a*** = [54.8 –6.04 0.166 0.121 –0.479]*^T^*. As a result, the calibration model takes the form of

(9) $$F(f_{0},h,a)=54.8-6.04f_{0}+0.166f_{0}^{2}+0.121h-0.479h^{2}$$

**Figure 11:**
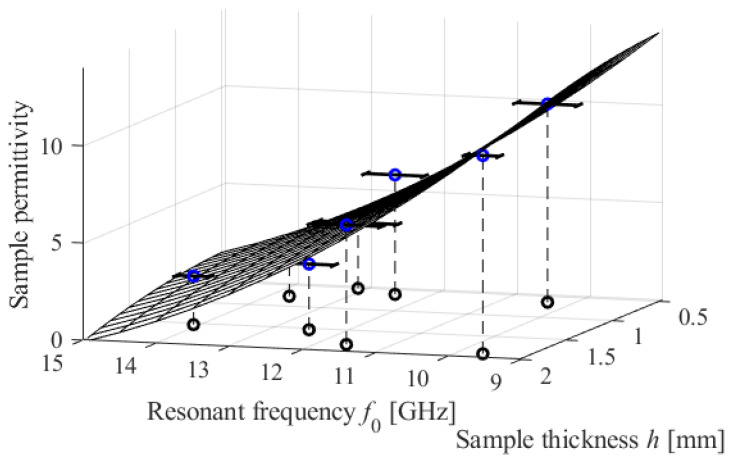
Inverse regression model (1) developed to calibrate the considered CCAR sensor. The surface depicts the model’s predictions as a function of the measured resonant frequency *f*_0_ of the sample, which is affected by its thickness *h*. The calibration samples are depicted in the form of blue circles. The horizontal bars in the graph depict the standard deviations of the recorded resonant frequency. These are calculated using data from ten separate measurement attempts.

Figure 11 shows a visual depiction of the model.

**Figure 12:**
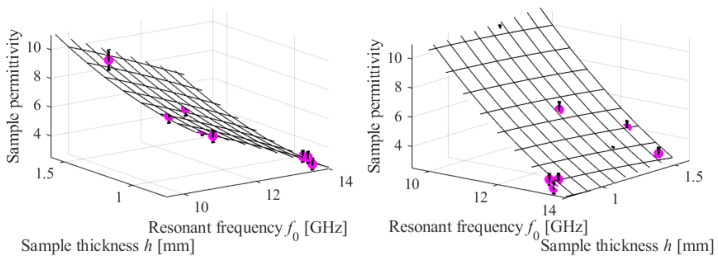
Experimental validation of the sensor and its calibration procedure. The verification samples are visually represented using circular shapes. Vertical bars represent the estimated permittivity prediction error, while the calibration model predictions are depicted by the surface. To enhance clarity, the data is presented for two distinct viewing perspectives.

The calibration technique and the sensor itself have been subjected to experimental verification utilizing six MUT samples, as outlined in Table 3. As stated, the inaccuracy in predicting permittivity is low, with an average relative difference of approximately five percent between the nominal and the predicted values. Moreover, the observed absolute prediction errors are within the expected bounds of prediction error. The latter is computed as

(10) $$d\varepsilon =\frac{\partial F(f_{0},h,a)}{\partial f_{0}}df_{0}$$

where *df*_0_ represents the estimated error of resonant frequency measurement (here, set to 0.25 GHz, based on the data in Table 2), whereas *∂F*/*∂f*_0_ is the sensitivity of the calibration model with respect to the resonant frequency at the frequency of measurement and the MUT’s thickness *h*. The verification samples are superimposed on the calibration model surface in Figure 12, together with the associated error bars.

It is important to highlight that the proposed methodology enables the calibration and subsequent employment of the sensor across wide spectra of the relative permittivity (ranging from 2.5 to 10.2) and thickness (the measurements range from around 0.5 mm to nearly 2.0 mm), with both parameters handled independently. 

The comparison between the CCAR microwave sensor and the existing state-of-the-art sensors is presented in Table 4, focusing on the resonant frequency, the calibration method, and relative sensitivity. 

The data shown in the table corroborates that the proposed resonator exhibits superior sensitivity while facilitating a fast and dependable calibration process. The latter utilizes an inverse regression model that incorporates both the permittivity and thickness of the sample.

## 5. Conclusions

This paper presented a newly developed complementary resonator that exhibits a high level of sensitivity. The primary objective of the resonator is to enable accurate and precise characterization of dielectric substrates, specifically in terms of their permittivity and thickness. The proposed configuration consists of a complementary crossed arrows resonator (CCAR) described by eight geometric parameters. The parameters have been tuned to attain a resonant frequency of 15 GHz. The optimized sensor has been manufactured on the AD250C substrate using LPKF Protolaser. Only a one percent relative discrepancy between the simulated and measured results for the unloaded sensor are observed. The fabricated sensor has been used to evaluate dielectric materials with thicknesses ranging from 0.5 mm to 2 mm and relative permittivity ranging from 2.5 to 10.2. Based on the obtained empirical data, an inverse regression model has been developed for the purpose of calibrating the CCAR sensor. The inverse model facilitates the direct estimation of the permittivity of a material being tested, based on its known thickness and the measured resonant frequency of the sample-loaded sensor. The CCAR sensor under consideration has a sensitivity of 5.74%, with a maximum measurement error of less than 8%, and an average error of only 3%. These parameters are comparable to those reported in the literature for the most advanced state-of-the-art metamaterial-based sensors. As well as its high performance, the important advantages of the presented device are its geometrical simplicity and low fabrication cost.

## Figures and Tables

**Table 1:** Reference samples used for sensor calibration.

Geometry of Structure	Fundamental Resonant Frequency (GHz/dB)	Quality Factor Unloaded	Resonant Frequency Due to Interaction with MUT	Frequency Shift (GHz)
CSSRR	8.6/−26.7	39	7.4/−27.5	1.2
M1	11.5/−25.6	41	9.8/−24.5	1.7
M2	9.1/−19.3	65	7.8/−16.3	1.3
M3	10/−23.6	46	8.5/−30.1	1.5
M4	12.9/−16.5	27	11.1/−14.1	1.8
CCAR (Pre-optimized)	13.2/−31.3	120	11.2/−32.5	2
CCAR (Optimized)	15/−46.2	136	12.6/−31.1	2.4

**Table 2:** Reference samples used for sensor calibration.

MUT	Relative Permittivity	Thickness (mm)	Average Resonant Frequency *f*_0_ (GHz)	Standard Deviation of Resonant Frequency (GHz)
AD250C	2.5	0.762	13.73	0.16
AD250C	2.5	1.524	14.08	0.23
RO4003C	3.38	0.508	13.11	0.35
RO4003C	3.38	1.545	12.50	0.23
RO4360	6.15	0.610	12.48	0.33
RO4360	6.15	1.83	11.60	0.16
RO3010	10.2	0.635	10.36	0.45
RO3010	10.2	1.905	9.64	0.20

**Table 3:** Sensor validation: Test samples and comparison of model-predicted and actual permittivity.

MUT	Nominal Relative Permittivity	Sample Thickness (mm)	Measured Resonant Frequency *f*_0_ (GHz)	Model-Predicted Permittivity (GHz)	Actual Prediction Error ^#^	Estimated Maximum Prediction Error ^$^
AD300C	2.97	1.524	13.41	2.73	0.24 [8%]	0.31
RO4003C	3.38	0.813	13.52	3.28	0.10 [3%]	0.30
RF-35	3.5	0.762	13.47	3.39	0.11 [3%]	0.31
FR-4	4.3	1.524	12.49	4.34	0.04 [1%]	0.37
RO4360	6.15	1.22	11.81	6.06	0.07 [1%]	0.42
RO3010	10.2	1.27	9.87	10.75	0.55 [5%]	0.55

^#^ Prediction error quantified as the difference between the calibration-model-predicted MUT permittivity and the nominal permittivity. The numbers in brackets represent relative error with respect to the nominal value, in percent. ^$^ Prediction error *dε* estimated as *dε* = (∂*F*/∂*f*_0_)*df*_0_, where *df*_0_ = 0.25 GHz is the assumed resonant frequency measurement error, whereas ∂*F*/∂*f*_0_ is the calibration model sensitivity corresponding to the measured resonant frequency and the thickness *h* of a given sample.

**Table 4:** Comparison with currently available high-tech microwave sensors.

Ref.	Resonator	Resonant Frequency (GHz)	Characterization Based on	Material under Test	Calibration Model	Relative Sensitivity
[23]	Symmetrical Split Ring Resonator	2.22	Permittivity	Rogers5880, Rogers 4350, FR4	Curve Fitting	1.51
[24]	Magnetic Resonator	1.65	Permittivity & Thickness	Rogers5880, Rogers 3006, Rogers 6010	No	3.19
[25]	Octagonal Spiral Resonator	2.48	Permittivity	PTFE, Rogers RO4350, F4BTM	Curve Fitting	4.61
[26]	Complementary Split Ring Resonator	4.1	Permittivity	Conductor Backed Dielectric	No	4.74
[27]	Electric Resonator	3.364	Permittivity & Thickness	Teflon, Polyethylene, Plexiglas, PVC, Dry Wood	Linear Fitting	4.91
[28]	Complementary Split Ring Resonator	14.45	Permittivity	TLY-5, AD300, RO4535, FR4	Inverse Regression Model	5.41
This Work	Complementary Crossed Arrows Resonator	14.85	Permittivity & Thickness	AD250C RO4003, FR4, R04360, RO3010	Inverse Regression Model	5.74

## Data Availability

Data are contained within the article.

## References

[1] Khan S., Marwat S.N.K., Khan M.A., Ahmed S., Gohar N., Alim M.E., Algarni A.D., Elmannai H. (2023). A self-decoupling technique to realize dense packing of antenna elements in MIMO arrays for wideband sub-6 GHz communication systems. Sensors.

[2] Cardillo E., Tavella F., Ampelli C. (2023). Microstrip Copper nanowires antenna array for connected microwave liquid sensors. Sensors.

[3] Monteagudo Honrubia M., Matanza Domingo J., Herraiz-Martínez F.J., Giannetti R. (2023). Low-cost electronics for automatic classification and permittivity estimation of glycerin solutions using a dielectric resonator sensor and machine learning techniques. Sensors.

[4] Pendry J.B., Holden A.J., Robbins D.J., Stewart W.J. (1999). Magnetism from conductors and enhanced nonlinear phenomena. IEEE Trans. Microw. Theory Tech..

[5] Falcone F., Lopetegi T., Baena J., Marwues R., Martin F., Sorolla M. (2004). Effective negative-epsilon stopband microstrip lines based on complementary split ring resonators. IEEE Microw. Wirel. Comp. Lett..

[6] Haq T., Ruan C., Zhang X., Kosar A., Ullah S. (2019). Low cost and compact wideband microwave notch filter based on miniaturized complementary metaresonator. Appl. Phys. A.

[7] Zhang J., Li C., Gao Y., Tan J., Xuan F., Ling X. (2022). Flexible multimode antenna sensor with strain and humidity sensing capability for structural health monitoring. Sens. Act. A Phys..

[8] Jorwal S., Dubey A., Gupta R., Agarwal D. (2023). A review: Advancement in metamaterial based RF and microwave absorbers. Sens. Act. A Phys..

[9] Deepti A., Gangwar D., Singh S., Sharma A., Singh S.P., Lay-Ekuakille A. (2021). Design of polarization conversion metasurface for RCS reduction and gain improvement of patch antenna for Ku-band radar sensing applications. Sens. Act. A Phys..

[10] Vélez P., Muñoz-Enano J., Grenier K., Mata-Contreras J., Dubuc D., Martin F. (2019). Split ring resonator-based microwave fluidic sensors for electrolyte concentration measurements. IEEE Sens. J..

[11] Acevedo-Osorio G., Reyes-Vera E., Lobato-Morales H. (2020). Dual-band microstrip resonant sensor for dielectric measurement of liquid materials. IEEE Sens. J..

[12] Yang X., Zhang M., Ren M., Mao S., Dhakal R., Kim N.Y., Cao Y., Li Y., Yao Z. (2023). Ultra-fast and high-sensitive tacrolimus solution detection based on microwave biosensor. Sens. Actuators A Phys..

[13] Haq T., Ruan C., Zhang X., Ullah S. (2019). Complementary metamaterial sensor for nondestructive evaluation of dielectric substrates. Sensors.

[14] Lee C.S., Yang C.L. (2014). Complementary split-ring resonators for measuring dielectric constants and loss tangents. IEEE Microw. Wirel. Comp. Lett..

[15] Han X., Li X., Zhou Y., Ma Z., Peng P., Fu C., Qiao L. (2022). Microwave sensor loaded with complementary curved ring resonator for material permittivity detection. IEEE Sens. J..

[16] Lee C.S., Yang C.L. (2015). Single-compound complementary split-ring resonator for simultaneously measuring the permittivity and thickness of dual-layer dielectric materials. IEEE Trans. Microw. Theory Tech..

[17] Lim S., Kim C.Y., Hong S. (2018). Simultaneous measurement of thickness and permittivity by means of the resonant frequency fitting of a microstrip line ring resonator. IEEE Microw. Wirel. Compon. Lett..

[18] Ji Q., Gu T., Gao Z., Yang M., Yuan Y., Du H., Wan F., Murad N.M., Fontgalland G., Silva H.S. (2023). CSRR DGS-based bandpass negative group delay circuit design. IEEE Access.

[19] Huang X. (2023). Design of miniaturized SIW filter loaded with improved CSRR structures. Electronics.

[20] Hu K.Z., Huang H.Y., Tang M.C., Chen Z., Yan D., Wang P. (2023). A single-layer wideband differential-fed filtering antenna with high selectivity. IEEE Trans. Antennas Propag..

[21] Bait-Suwailam M.M. (2023). Towards monitoring and identification of red palm weevil gender using microwave CSRR-loaded TL sensors. Sensors.

[22] Haq T., Koziel S. (2023). Rapid design optimization and calibration of microwave sensors based on equivalent complementary resonators for high sensitivity and low fabrication tolerance. Sensors.

[23] Alahnomi R.A., Zakaria Z., Ruslan E., Ab Rashid S.R., Mohd Bahar A.A. (2017). High Q sensor based on symmetrical split ring resonator with spurlines for solid material detection. IEEE Sens. J..

[24] Ebrahimi A., Scott J., Ghorbani K. (2020). Dual-mode resonator for simultaneous permittivity and thickness measurement of dielectrics. IEEE Sens. J..

[25] Liu Q., Deng H., Meng P., Sun H. (2021). High sensitivity sensor loaded with octagonal spiral resonators for retrieval of solid material permittivity. IEEE Sens. J..

[26] Alharbi F.T., Haq M., Udpa L., Deng Y. (2022). Characterization of conductor-backed dielectric substrates using a novel resonance-based method. IEEE Sens. J..

[27] Varshney P.K., Kapoor A., Akhtar M.J. (2021). Highly sensitive ELC resonator based differential sensor. IEEE Trans. Instrum. Meas..

[28] Haq T., Koziel S. (2022). Inverse modeling and optimization of CSRR-based microwave sensors for industrial applications. IEEE Trans. Microw. Theory Tech..

